# Membrane stripping in group B streptococcus carriers does not impede adequate intrapartum antibiotic prophylaxis: a retrospective study

**DOI:** 10.3389/fmed.2024.1368998

**Published:** 2024-04-05

**Authors:** Doron Kabiri, Ora Paltiel, Noa Ofek-shlomai, Ran Nir-Paz, Yishai Sompolinsky, Yossef Ezra

**Affiliations:** ^1^Department of Obstetrics and Gynecology, Hadassah Hebrew Medical Center and Faculty of Medicine, Hebrew University of Jerusalem, Jerusalem, Israel; ^2^Braun School of Public Health and Community Medicine, Hadassah Hebrew Medical Center and Faculty of Medicine, Jerusalem, Israel; ^3^Department of Neonatology, Hadassah-Hebrew University Medical Center and Faculty of Medicine, Jerusalem, Israel; ^4^Department of Clinical Microbiology and Infectious Diseases, Hadassah Hebrew Medical Center and Faculty of Medicine, Jerusalem, Israel

**Keywords:** group B streptococcus, intrapartum antibiotic prophylaxis, membrane stripping, labor, pregnancy, primiparity, multiparity, maternal and neonatal outcomes

## Abstract

**Objective:**

Membrane stripping in group B streptococcus (GBS) carriers poses an increased risk of inadequate antibiotic prophylaxis, potentially due to accelerated labor, thereby potentially impacting the management of GBS colonization during delivery. We compared the adequacy of intrapartum antibiotic prophylaxis between pregnant women colonized with GBS, who underwent membrane stripping and those who did not. The study aimed to determine whether the performance of membrane stripping, by potentially shortening labor duration, increases the risk of inadequate antibiotic prophylaxis dispensation.

**Study design:**

A retrospective cohort study was conducted on GBS screen-positive women with a full-term singleton pregnancy in cephalic presentation, who were eligible for vaginal delivery. The exposed group consisted of women who underwent membrane stripping, while the unexposed group consisted of women who did not undergo membrane stripping. The primary outcome was defined as inadequate duration of antibiotic prophylaxis during labor, wherein less than 4 h of beta-lactam antibiotics were administered prior to delivery. Neonatal outcome was compared between the groups.

**Results:**

This retrospective cohort study comprised 1,609 women, with 129 in the exposed group (stripping group) and 1,480 in the unexposed group (no stripping group). Adequate intrapartum antibiotic prophylaxis was received by 64.3% (83/129) of the exposed group, compared to 46.9% (694/1,480) of the unexposed group (*p* = 0.003). Membrane stripping was associated with increased odds of receiving adequate prophylaxis (OR 1.897, 95% CI 1.185–3.037, *p* = 0.008). After excluding women who presented to the labor ward in active labor and delivered in less than 4 h, both the exposed and unexposed groups had similarly high rates of adequate intrapartum antibiotic prophylaxis (87.5% vs. 85.8%, respectively). No significant difference was observed in adverse neonatal outcomes between the groups.

**Conclusion:**

The provision of membrane stripping did not impede adequate intrapartum antibiotic prophylaxis and was correlated with a higher rate of sufficient prophylaxis in comparison to non-swept patients. These observations suggest that membrane stripping can be considered a safe option for ensuring adequate antibiotic prophylaxis in women colonized with GBS.

## Introduction

Early onset neonatal group B streptococcus (GBS) disease is characterized by the onset of infection within the first 7 days of life, presenting usually within the initial 24–48 h (1). Its clinical manifestations range from mild illness to severe respiratory disease, meningitis, and neonatal sepsis. Late-onset neonatal GBS disease occurs after the 7th day of life and is characterized by clinical signs of bacteremia, meningitis, and other focal infections, such as osteomyelitis, septic arthritis, skin and soft tissue infections, urinary tract infections, and pneumonia (2, 3).

Since the 1980s, nationwide population-based surveillance studies have consistently demonstrated that GBS screening and subsequent intrapartum antibiotic prophylaxis (IAP) reduce vertical transmission and early-onset disease (4–8). In the 1990s, the United States initiated national surveillance and recommended strategies for identifying GBS colonized pregnant women who are suitable for IAP. The Centers for Disease Control and Prevention (CDC) published guidelines in 1996 that suggested either risk-based or screening-based approaches for preventing neonatal GBS disease. The incidence of early-onset GBS disease significantly decreased by nearly 70% from 1.7 cases per 1,000 live births in 1993 to 0.6 cases per 1,000 live births in 1998 (9, 10). Subsequent updates to the national guidelines in 2002 recommended universal antenatal screening of all pregnant women at 35–37 weeks of gestation using vagino-rectal swabs. Rapid adoption of these guidelines led to increased administration of IAP to GBS-colonized women, which resulted in further decreases in early-onset GBS neonatal disease from 0.47 cases per 1,000 live births in 1999–2004 to 0.32 cases per 1,000 live births (6, 7, 11–19).

*Streptococcus agalactiae*, commonly known as group B streptococcus (GBS), is a gram-positive coccus that is facultatively anaerobic and commonly found in chains or diplococci. It is predominantly found in the gastrointestinal and genital tracts, and approximately 10%–30% of gravid women are carriers of GBS (1, 11, 20, 21). The maternal colonization of GBS is influenced by various risk factors, such as poor local hygiene, sexual activity, obesity, Afro-American ethnicity, the use of tampons or intrauterine devices, and the absence of lactobacilli in the gastrointestinal tract (20). Neonatal colonization and disease primarily occur due to maternal colonization and the bacterium can be transmitted during labor through the birth canal or via ascending infection, or aspiration of infected amniotic fluid (although the latter is less common). GBS is the sole origin of early-onset neonatal GBS disease (11), and can lead to maternal urinary tract infection, bacteremia, endometritis, chorioamnionitis, and wound infection (1, 7, 22, 23). The risk factors for neonatal colonization and disease include maternal colonization, prolonged rupture of membranes, gestational age less than 37 weeks, chorioamnionitis, maternal bacteriuria during the current pregnancy, previous delivery of an infant with GBS disease, young maternal age, low levels of maternal anti-GBS antibodies, black ethnicity, and fetal male sex (1, 7, 20, 24, 25).

Membrane stripping is a long-standing and uncomplicated procedure for labor induction, initially reported in 1810 by Hamilton in England. This process entails separating the chorioamniotic membranes manually from the internal orifice of the uterine cervix by circular motion of the examiner’s finger during a vaginal examination. This technique initiates a sequence of physiological responses, wherein local production of prostaglandins results in uterine contractions and facilitates cervical ripening. Membrane stripping performed at term has been associated with a reduction in the need for formal induction of labor and a shorter time until the onset of spontaneous labor, resulting in a decrease in the incidence and risks of post-term pregnancies. The most frequently reported adverse effects of the procedure include temporary maternal discomfort, minor bleeding, and irregular contractions. A recent Cochrane review has confirmed that membrane stripping does not increase the incidence of maternal and neonatal infection (26–29).

Despite the findings of this Cochrane review (26), controversy persists regarding performing membrane stripping in GBS carriers. Due to the theoretical risk of bacterial seeding and concerns regarding fast labors and inadequate antibiotic prophylaxis following the procedure, some practitioners refrain from performing the procedure in GBS colonized patients (30, 31). The Centers for Disease Control and Prevention (CDC) and the American College of Obstetricians and Gynecologists (ACOG) have noted that the available data are insufficient to definitively guide the decision of whether or not to perform membrane stripping in GBS carriers (7, 11, 30). Consequently, some practitioners elect not to perform the procedure in these patients.

Owing to the high prevalence of GBS colonization, the paucity of data regarding the safety of membrane stripping in GBS carriers hinders a significant proportion of obstetrical patients from receiving optimal obstetric care. A critical component of appraising the safety of this intervention in these patients is determining whether it hastens labor in a manner that compromises the adequacy of antibiotic prophylaxis, which must be administered no less than 4 h before delivery (6, 7, 32, 33). A study performed by Van Dyke et al. (6) demonstrated that a substantial majority of GBS carriers received adequate prophylactic treatment during labor, though the study did not distinguish between those who underwent membrane sweeping and those who did not.

The present study aimed to compare the adequacy of antibiotic treatment in pregnant women colonized with GBS who underwent membrane sweeping versus those who did not. The primary objective was to examine whether membrane sweeping in GBS carriers poses an increased risk of inadequate antibiotic prophylaxis, thereby potentially impacting the management of GBS colonization during delivery.

## Materials and methods

### Study design and participants

This retrospective cohort study was conducted at a tertiary university-affiliated medical center, and aimed to investigate the adequacy of antibiotic prophylaxis in GBS-positive women who underwent singleton-term labor. Trained personnel extracted demographic information, along with medical, prenatal, and antenatal history, from patient medical records. The study population was comprised of GBS-positive women who gave birth at the hospital during a 27 months period. The study was approved by the institutional review board (IRB No.: 0204-11-HMO).

The present study recruited women aged 18 to 40 years who tested positive for GBS colonization and had singleton-term pregnancies with cephalic presentation suitable for vaginal delivery. Participants who underwent membrane sweeping were categorized into the exposed group, while those who did not receive the procedure comprised the unexposed group. Women with contraindications to vaginal delivery, multiple gestations, significant fetal anomalies, evidence of intrauterine infections, or who declined intrapartum chemoprophylaxis were excluded from the study.

### Treatment protocol

Throughout the study period, women who met the inclusion criteria and were scheduled for vaginal delivery were provided with the opportunity to undergo membrane sweeping, as per the National Institute for Health and Care Excellence (NICE) recommendations (28). The procedure was carried out by trained obstetric staff after confirming the fetal position, the location of the placenta, and biophysical profile by ultrasonography, in addition to fetal well-being as assessed by non-stress testing, with the patient’s consent. Following the procedure, patients were discharged and continued routine obstetric care following standard protocol, which included regular non-stress and biophysical profile testing. Labor induction was considered for patients who reached 42 weeks of gestation or in cases of maternal or fetal indications. We did not routinely assess Bishop scores for women who did not undergo membrane stripping; hence, we cannot make direct comparisons of Bishop scores between the groups at the time of the proposal for membrane sweeping.

In line with the established CDC recommendations (7), intrapartum chemoprophylaxis for GBS is advised for women with positive recto-vaginal cultures, prior history of invasive GBS disease in the infant during delivery, or confirmed GBS bacteriuria during pregnancy. Penicillin is the first-line chemoprophylaxis regimen for intrapartum GBS management, with ampicillin as an acceptable alternative. If women have a documented penicillin allergy but no history of severe anaphylaxis, angioedema, respiratory distress, or urticaria, cefazolin is administered. For those at high risk of severe anaphylaxis, clindamycin or vancomycin may be considered, depending on the resistance profile of the isolated GBS strain. In cases where clinical suspicion of chorioamnionitis is present, defined as the presence of maternal fever greater than 38°C/100.4°F or foul-smelling amniotic fluid, appropriate diagnostic measures including blood and urine cultures were taken. If necessary, broad-spectrum antibiotics were initiated.

### Outcomes

The primary outcome measure of this study was the rate of full intrapartum antibiotic prophylaxis administration, defined as the administration of antibiotics for a minimum duration of 4 h preceding delivery. The adequacy of antibiotic prophylaxis was ascertained based on the administration of beta-lactam antibiotics for a minimum of 4 h before delivery (34). Conversely, insufficient antibiotic prophylaxis was defined as the administration of antibiotics for less than 4 h prior to delivery, following established guidelines (7, 32, 33). The secondary outcome measures included neonatal morbidities, including birthweight Apgar scores ≤7, days in NICU, and incidence of early or late-onset neonatal GBS disease. The neonatal follow-up in our study extended throughout the entire duration of the neonatal hospitalization, from delivery until discharge.

### Statistical methods

The sample size calculation was based on detecting a clinically negligible difference in the percentage of women receiving adequate antibiotic prophylaxis between the study and control groups. The ratio of women who underwent membrane sweeping vs. those who did not was assumed to be 1:3, with a one-sided significance level of 5%, an 80% power, a common proportion of 87% of adequate prophylaxis in the population (6, 7), and a 10% clinically negligible difference. As a result, a sample size of at least 114 women in the membrane sweeping group and 341 in the control group was found to be appropriate for testing the hypothesis. Statistical analysis was conducted using the chi-square test and Fisher’s exact test for categorical variables, ANOVA for continuous variables, and t-test for a categorical variable with two categories and a quantitative variable. A *p*-value of less than 0.05 was considered statistically significant.

## Results

**Figure 1:**
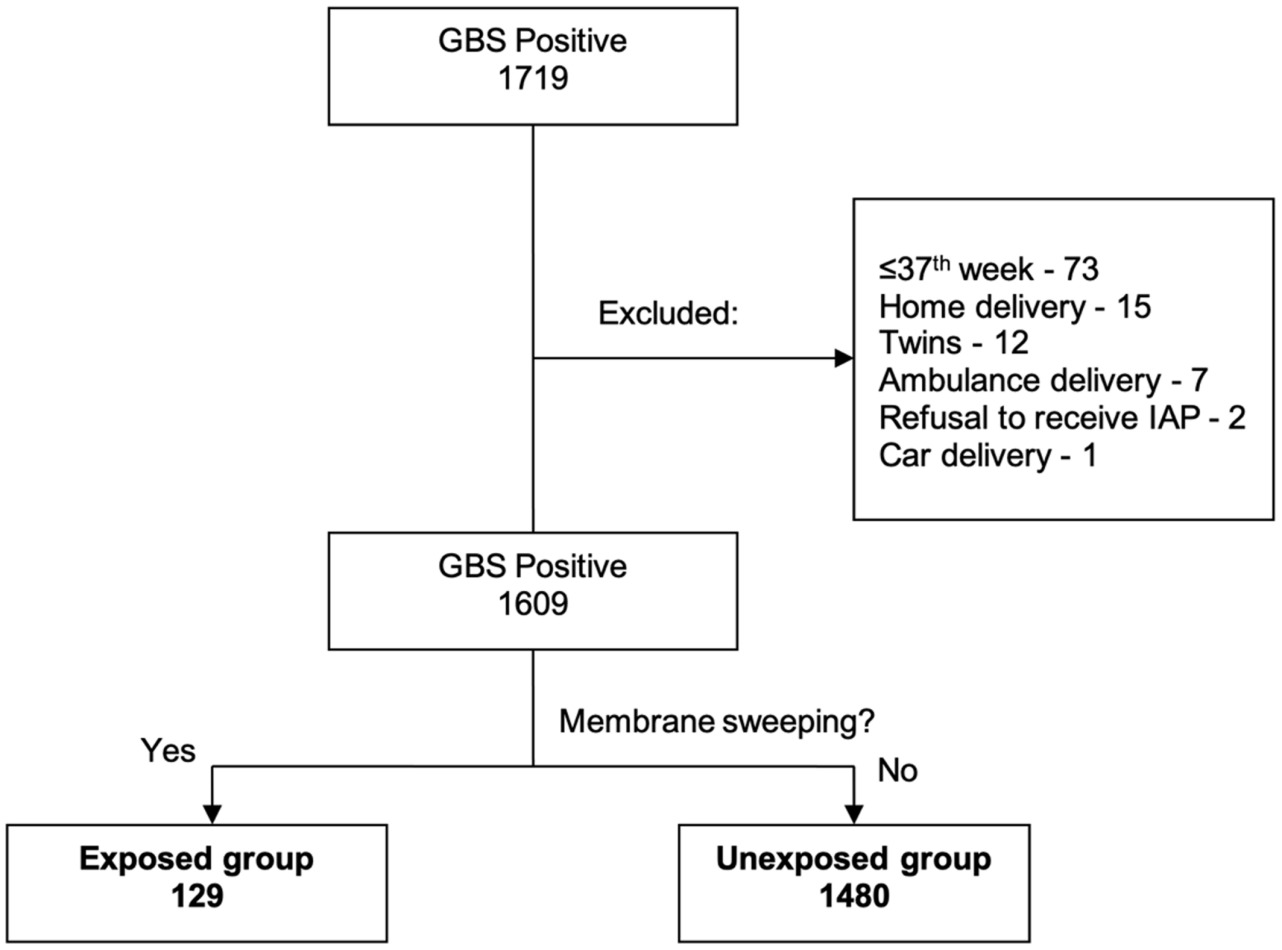
Participant screening and allocation to the study groups. Flowchart of participant selection and group allocation in the study.

During the study period, a total of 7,681 gravid women were identified as eligible for vaginal delivery at our tertiary care center. Of these, 1,719 (22.3%) were confirmed positive for GBS screening. Following exclusion of 184 subjects based on predetermined criteria, a final sample of 1,609 qualified women remained (Figure 1). Membrane sweeping was performed in 129 (8%) and comprised the exposed group, while 1,480 (92%) did not undergo the procedure and were designated the unexposed group. The groups were comparable regarding their demographic and obstetric parameters, as shown in Table 1. The difference in the mean gestational age at labor between the two groups was statistically significant.

**Table 1:** Demographic and obstetric characteristics at labor.

Characteristics	Exposed group (*N* = 129) 8%	Unexposed group (*N* = 1,480) 92%	*p*-value
Maternal age (years)	30.89 ± 5.47	30.09 ± 5.17	0.094
Gestational age at labor (weeks) (mean ± SD)	39.82 ± 1.01	39.62 ± 1.03	0.033
Gravidity (*N*) (mean ± SD)	3.24 ± 2.36	3.25 ± 2.32	0.946
Parity (*N*) (mean ± SD)	1.81 ± 1.97	1.82 ± 1.94	0.954
Abortions (*N*) (mean ± SD)	0.42 ± 0.82	0.43 ± 0.82	0.875
Living children (*N*) (mean ± SD)	1.79 ± 1.98	1.82 ± 1.95	0.866
Previous cesarean delivery (*N*)	13 (10.1%)	120 (8.1%)	0.436
Primiparous (*N*)	36 (27.9%)	423 (28.6%)	0.871
Multiparous (*N*)	93 (72.1%)	1,057 (71.4%)	

Table 2 presents the intrapartum characteristics of the study participants. There were significant differences between the exposed and unexposed groups. A greater proportion of women in the exposed group (59.7%) who underwent membrane sweeping had a more advanced cervical status, with a Bishop score greater than 5, while most women in the unexposed group (52.6%) had a simplified Bishop score of 5 or less (*p* = 0.008). The exposed group had a lower rate of labor induction initiation (10.1% vs. 18.6%, *p* = 0.015) and a higher rate of epidural anesthesia administration (66.7% vs. 50%, *p* < 0.001). Despite presenting with a more advanced cervical stage, the mean duration in hours from 4 cm dilatation (defined as the active stage of delivery) to delivery was longer in the exposed group (4.51 ± 4.02 vs. 3.34 ± 3.22 h).

**Table 2:** Intrapartum characteristics.

Characteristics	Exposed group (*N* = 129) 8%	Unexposed group (*N* = 1,480) 92%	*p*-value
Simplified Bishop score at admission to labor ≤5^a^	52 (40.3%)	778 (52.6%)	0.008
*Mode of delivery*			
Vaginal	104 (89.6%)	1,217 (82.2%)	0.572
Instrumental	11 (8.5%)	141 (9.5%)	
Emergent CD	14 (10.9%)	122 (8.2%)	
*Initiation of labor*			
Spontaneous	106 (89.9%)	1,204 (81.4%)	0.015
Induction	13 (10.1%)	276 (18.6%)	
Hours from 4 cm dilatation to delivery	4:51 ± 4:02	3:34 ± 3:22	0.001
Less than 4 h from 4 cm dilatation to delivery	65 (50.4%)	965 (65.2%)	0.001
Hours from PROM to delivery	5:19 ± 9:57	5:49 ± 10:05	0.583
Epidural	86 (66.7%)	740 (50%)	<0.001
Intrapartum fever	4 (3.1%)	41 (2.8%)	0.779
Male gender	66 (51.2%)	725 (49.0%)	0.635
Female gender	63 (48.8%)	755 (51.0%)	
Birth weight (grams)	3,521 ± 409	3,341 ± 399	<0.001
Apgar at 5th minute <7	0 (0.0%)	1 (0.01%)	>0.999
NICU admission	1 (0.8%)	3 (0.2%)	0.284

CD, cesarean delivery; Cm, centimeter; PROM, premature rupture of membranes; NICU, neonatal intensive care unit.

a “Simplified Bishop score” was calculated according to the article of Laughon et al. (19).

### Primary outcome

**Figure 2:**
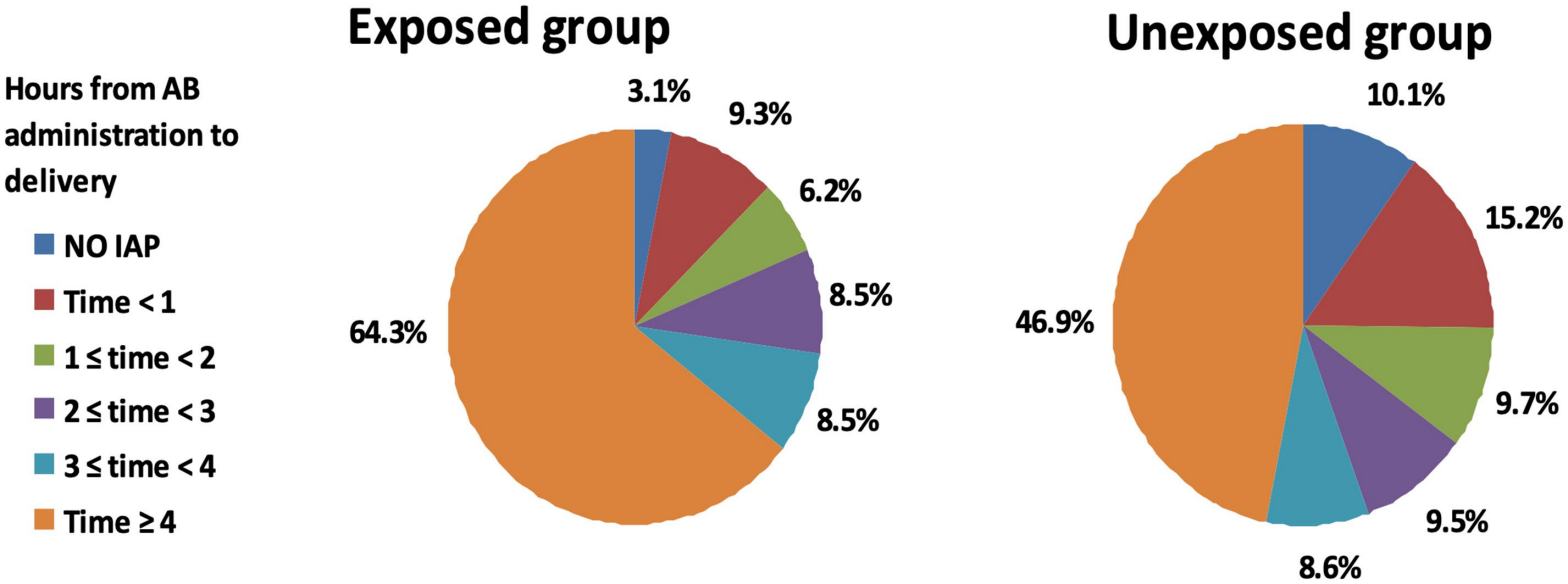
Comparison of intrapartum antibiotic prophylaxis duration between exposed and unexposed groups in GBS positive women. Pie charts depicting the duration of intrapartum antibiotic prophylaxis across the study groups. The left pie chart represents the exposed group that underwent membrane stripping, while the right pie chart corresponds to the unexposed group that did not undergo the procedure. Each segment of the pie charts reflects the proportion of patients by the length of antibiotic prophylaxis received, with colors corresponding to different durations as indicated in the legend.

The primary outcome of the study was the ratio of women receiving adequate intrapartum antibiotic prophylaxis, which was defined as the administration of beta-lactam antibiotics for at least 4 h before delivery. Among the women in the Exposed group, 64.3% (83/129) obtained adequate prophylaxis, while 46.9% (694/1,480) of those in the non-membrane sweeping group received adequate prophylaxis (Figure 2 and Table 3). The difference in percentages between the exposed and unexposed groups was statistically significant (*p* = 0.003).

**Table 3:** Duration of intrapartum antibiotic prophylaxis by the study groups (*p* = 0.003).

Time from AB administration to delivery (hours)	Exposed group (*N* = 129)	Unexposed group (*N* = 1,480)	Total
No IAP	4 (3.1%)	149 (10.1%)	153 (9.5%)
Time < 1	12 (9.3%)	225 (15.2%)	237 (14.7%)
1 ≤ time < 2	8 (6.2%)	144 (9.7%)	152 (9.4%)
2 ≤ time < 3	11 (8.5%)	141 (9.5%)	152 (9.4%)
3 ≤ time < 4	11 (8.5%)	127 (8.6%)	138 (8.6%)
Time ≥ 4	83 (64.3%)	694 (46.9%)	777 (48.3%)

AB, antibiotic; IAP, intrapartum antibiotic prophylaxis.

In sub-analysis, following the exclusion of women who presented in the active stage of labor (less than 4 h from admission to delivery), we noted that a high proportion of women in both the exposed and unexposed groups received adequate intrapartum antibiotic prophylaxis, with rates of 87.5 and 85.8%, respectively (Table 4).

**Table 4:** Duration of intrapartum antibiotic prophylaxis for women with an active stage of 4 h or more from 4 cm dilatation to delivery (*p* = 0.983).

Time from AB administration to delivery (hours)	Exposed group with an active phase of ≥4 h (*N* = 64) 49.6%	Unexposed group with an active phase of ≥4 h (*N* = 515) 34.8%	Total
No IAP	1 (1.6%)	10 (1.9%)	11 (1.9%)
Time < 1	0 (0.0%)	8 (1.6%)	8 (1.4%)
1 ≤ time < 2	1 (1.6%)	7 (1.4%)	8 (1.4%)
2 ≤ time < 3	1 (1.6%)	11 (2.1%)	12 (2.1%)
3 ≤ time < 4	5 (7.8%)	37 (7.2%)	42 (7.3%)
Time ≥ 4	56 (87.5%)	442 (85.8%)	498 (86.0%)

AB, antibiotic; IAP, intrapartum antibiotic prophylaxis.

After comparing the duration from the active stage of labor to delivery between primiparous and multiparous women, we observed a statistically significant longer duration of the first delivery, as expected. However, we did not observe any statistically significant differences in subsequent deliveries (Table 5).

**Table 5:** Duration of labor.

	Less than 4 h from 4 cm dilatation to delivery (*N* = 1,030)	4 h or more from 4 cm dilatation to delivery (*N* = 579)	*p*-value
Primiparous	172 (37%)	287 (63%)	<0.001
Multiparous	858 (75%)	292 (25%)	

H, hours; Cm, centimeter.

In this study, we identified certain variables that significantly correlated with receiving adequate intrapartum antibiotic prophylaxis (Table 6). Specifically, 334 (72.7%) of primiparous women and 498 (60%) of those with a simplified Bishop score ≤5 at labor admission, received adequate antibiotic prophylaxis. This could be attributed to a prolonged active stage of labor, with a mean duration of 5:30 ± 3:43 h, which provided a window of opportunity for administering antibiotics for at least 4 h before delivery.

**Table 6:** Background factors associated with adequate intrapartum antibiotic prophylaxis.

Characteristics	Adequate IAP (*N* = 777) 48.3%	Inadequate IAP (*N* = 832) 51.7%	*p*-value
Maternal age (year)	29.81 ± 5.20	30.48 ± 5.17	0.010
Gestational age at labor (weeks)	39.66 ± 1.05	39.62 ± 1.01	0.482
Gravidity	2.82 ± 2.30	3.66 ± 2.65	<0.001
Parity	1.41 ± 1.89	2.21 ± 1.91	<0.001
Abortions	0.4 ± 0.79	0.46 ± 0.84	0.165
Living children	1.40 ± 1.89	2.21 ± 1.93	<0.001
Previous CD	67 (50.3%)	66 (49.7%)	0.615
Primiparity	334 (72.7%)	125 (27.3%)	<0.001
Simplified Bishop score at admission to labor ≤5^a^	498 (60.0%)	332 (40.0%)	<0.001
Induction of labor	229 (79.2%)	60 (20.8%)	<0.001
Hours from 4 cm dilatation to delivery	5:30 ± 3:43	1:58 ± 2:02	<0.001
Membrane stripping	83 (64.3%)	46 (35.7%)	<0.001
No membrane stripping	694 (46.9%)	786 (53.1%)	
Hours from PROM to delivery	9:33 ± 11:31	2:15 ± 6:49	<0.001
Epidural	564 (68.3%)	262 (31.7%)	<0.001
Male gender	394 (49.8%)	397 (50.2%)	0.230
Birth weight (grams)	3,355 ± 413	3,355 ± 392	0.991
Apgar at 5th minute <7	0 (0.0%)	1 (100.0%)	>0.999
NICU admission	3 (75.0%)	1 (25.0%)	0.358

IAP, intrapartum antibiotic prophylaxis; CD, cesarean delivery; Cm, centimeter; PROM, premature rupture of membranes; NICU, neonatal intensive care unit.

a “Simplified Bishop score” was calculated according to the article of Laughon et al. (19).

Regarding our primary hypothesis, we observed a greater proportion of women who underwent membrane sweeping received sufficient intrapartum antibiotic prophylaxis compared to their non-swept counterparts [83/129 (64.3%) vs. 694/1,480 (46.9%), respectively]. Our multivariable logistic regression analysis (Table 7) demonstrated that even after controlling for confounding factors, membrane sweeping was associated with a higher adjusted odds ratio of receiving adequate intrapartum antibiotic prophylaxis (aOR = 1.897, 95% CI 1.185–3.037, *p*-value 0.008).

**Table 7:** Factors associated with adequate intrapartum antibiotic prophylaxis (multiple logistic regression analysis).

Characteristics	Adjusted OR	95% confidence interval	*p*-value
Maternal age (years)	1.029	1.001–1.057	0.040
Primiparity	1.999	1.433–2.788	<0.001
Simplified Bishop score at admission to labor ≤5^a^	1.869	2.466–1.417	<0.001
Induction of labor	5.129	7.433–3.540	<0.001
Epidural	2.272	2.980–1.731	<0.001
Hours from 4 cm dilatation to delivery	1.669	1.784–1.560	<0.001
Membrane sweeping	1.897	1.185–3.037	0.008

a “Simplified Bishop score” was calculated according to the article of Laughon et al. (19).

A detailed examination of the neonatal outcomes was performed, focusing on the immediate postnatal period and subsequent hospital stay. In the GBS screening-positive group, adverse neonatal outcomes were observed in 8 out of 129 neonates, representing a rate of 6.2%. These outcomes ranged from minor health concerns such as hyperbilirubinemia and transient tachypnea of the newborn, to more significant morbidities requiring neonatal intensive care unit admission. There was no statistically significant difference in the rate of adverse neonatal outcomes between the GBS screening-positive group who underwent membrane stripping and the other groups, indicating that membrane stripping in GBS carriers does not lead to an increase in neonatal complications.

## Discussion

In this retrospective cohort study of 1,609 women with GBS colonization, membrane sweeping was significantly associated with an increased rate of adequate intrapartum antibiotic prophylaxis (64.3% vs. 46.9%, adjusted OR 1.897, 95% CI 1.185–3.037, *p* = 0.008) in the exposed group compared to the unexposed group. However, after excluding women admitted in active labor and who delivered after less than 4 h, both groups received equivalently high rates of adequate prophylaxis (87.5 and 85.8%, respectively).

Membrane sweeping is a common obstetric intervention aimed at expediting the onset of labor. A recent Cochrane review (26) confirmed its safety but did not address the consequences of membrane sweeping for group B Streptococcus (GBS) carriers. Despite the fact that GBS carriage rates in pregnant women vary from 10–30%, the safety of membrane sweeping in these women remains undetermined due to inadequate evidence (7, 11, 30). Consequently, many clinicians still avoid performing this procedure on GBS carriers.

A previous investigation conducted by our research team (35) demonstrated no significant difference in maternal or neonatal outcomes between patients who were GBS-positive and those who had negative or unknown GBS status after undergoing membrane sweeping. Further, it’s important to note that all neonates included in the study were monitored closely, with a particular focus on the signs of early-onset neonatal sepsis - a potentially severe complication related to GBS infection. There were no cases of early-onset neonatal sepsis observed in our study. Therefore, our findings indicate that even when membrane stripping is undertaken in GBS-positive women, it does not increase the rate of adverse neonatal outcomes.

An additional study by Van Dyke et al. (6), showed that 87% of women with GBS colonization received adequate antibiotic prophylaxis during delivery. However, this study did not investigate the potential impact of membrane sweeping on prophylaxis adequacy or consider the subgroup of women who had a prolonged active phase of labor lasting at least 4 h.

One possible explanation for the greater adequacy of intrapartum antibiotic prophylaxis in the exposed group may be linked to the thorough explanation given to patients about the outcomes of membrane sweeping and the significance of antibiotic treatment before the procedure. This could have heightened their awareness and prompted them to arrive at the hospital earlier in labor, enabling timely administration of antibiotics.

Additional factors associated with higher rates of adequate treatment were observed, including primiparity (adjusted odds ratio (aOR) 1.999), Bishop score ≤5 at admission to the labor ward (aOR 1.869), and a prolonged active stage of labor, which was defined as the duration from 4 cm dilatation to delivery (aOR 1.669). These factors could lead to a longer duration of labor and delivery, which may have enabled healthcare providers to administer antibiotics effectively.

The association between multiparity and shorter labor duration is widely recognized and may lead to increased rates of suboptimal intrapartum antibiotic prophylaxis. Our investigation revealed that 75% of multiparous women had an active stage of labor less than 4 h, in contrast to only 25% of primiparous women. This outcome should be weighed when considering the use of membrane sweeping in parous GBS carriers.

Based on the results of our study, which showed that the majority of GBS carriers who underwent membrane sweeping received adequate intrapartum antibiotic prophylaxis, and that there was no increased risk of adverse effects compared to the general population, we can infer that membrane sweeping for GBS carriers is an innocuous intervention and a safe procedure.

### Strengths and limitations

The interpretation of our study results should take into account both its strengths and limitations. The study’s considerable sample size of 1,609 women allowed for a thorough analysis of the association between membrane sweeping and the adequacy of intrapartum antibiotic prophylaxis in GBS carriers. The employment of rigorous statistical techniques and multiple sub-analyses to control for potential confounding variables and identify factors linked to higher rates of adequate treatment, bolster the validity of our results.

Notwithstanding its significant strengths, our study has several noteworthy limitations that need to be considered. The retrospective nature of the study design may have led to biases, as compared to a prospective study design. Nonetheless, the large number of participants in our study helped mitigate some of these biases that are inherent in retrospective studies. Additionally, the delivery of patients was managed by multiple healthcare providers, such as midwives and physicians, potentially introducing heterogeneity in the treatment methods.

### Future research

While our study has provided significant contributions to the understanding of the safety and efficacy of membrane sweeping in GBS carriers, there remain certain areas that require further investigation. To validate our findings and to examine the generalizability of our results to other populations, a multicenter trial involving a heavily GBS colonized population would strengthen our results. In addition, future research could explore the most appropriate timing of membrane sweeping and intrapartum antibiotic prophylaxis in GBS carriers, as well as their effects on maternal and neonatal outcomes.

## Conclusion

To conclude, our study demonstrates that the use of membrane sweeping in GBS carriers does not lead to suboptimal intrapartum antibiotic prophylaxis adequacy. The results also suggest that the procedure may even have a positive effect, with higher rates of adequate prophylaxis compared to GBS carriers who did not undergo membrane sweeping. This finding may be attributed to the enhanced awareness of both patients and medical practitioners regarding the significance of antibiotic treatment after the procedure. Although the retrospective nature of our research presents a potential challenge, the extensive sample size and meticulous statistical analysis lend support to our conclusions.

## Data availability statement

The raw data supporting the conclusions of this article will be made available by the authors, without undue reservation.

## Ethics statement

The studies involving humans were approved by Hadassah Institutional Review Board (IRB No.: 0204-11-HMO). The studies were conducted in accordance with the local legislation and institutional requirements. Written informed consent for participation was not required from the participants or the participants’ legal guardians/next of kin in accordance with the national legislation and institutional requirements.

## Author contributions

DK: Conceptualization, Data curation, Formal analysis, Funding acquisition, Investigation, Methodology, Project administration, Resources, Software, Supervision, Validation, Visualization, Writing – original draft, Writing – review & editing. OP: Conceptualization, Data curation, Writing – review & editing, Formal analysis, Investigation, Supervision, Validation, Visualization, Writing – original draft. NO-s: Conceptualization, Data curation, Writing – review & editing. RN-P: Conceptualization, Data curation, Writing – review & editing. YS: Data curation, Writing – review & editing. YE: Conceptualization, Data curation, Project administration, Supervision, Validation, Writing – original draft.
